# Environmental Tobacco Smoke: Incomplete Research or Author Bias?

**DOI:** 10.1289/ehp.10233

**Published:** 2007-09

**Authors:** Roger A. Jenkins

**Affiliations:** Consultant, Bozeman, Montana, E-mail: mcdonaldjenkins@twohikers.org

As primary author and co-principal investigator of the 16 Cities Study (Jenkins et al. 1996), I was disappointed to read the article by Barnes et al. (2006). Their methodology, relying solely on Internet-based searching of trial and hearing testimony, tobacco industry documents, and other information, was clearly incomplete and suggests poor investigatory skills and/or deliberate selection of data or information to support a preconceived notion of the complex processes involved with the conception, conduct, data reporting, and interpretation of what became known as the 16 Cities Study.

One example (there are many) of the errors presented by Barnes et al. (2006) is the paragraph on page 1895 devoted to highlighting the fact that in the Broin flight attendant class action lawsuit, Judge Robert Kaye ruled (mistakenly I believe) that I could not rely upon the 16 Cities Study in my testimony for that case. What the authors failed to mention is that in the Dunn-Wiley environmental tobacco smoke (ETS) trial in Muncie, Indiana, less than 6 months later (Dunn and Wiley v. RJR Nabisco Holdings Corp, et al. 1998), Delaware Superior Court 1 Judge Robert Barnet Jr. overruled a similar motion by the plaintiffs, and noted that he did not find the motion well taken. Did Barnes et al. simply miss that ruling in their search for information, or did they ignore it?

Probably the most egregious distortions of the facts lies in the claims of Barnes et al. (2006) that we failed to make the Occupational Safety and Health Administration (OSHA) or the public aware of RJ Reynolds’ involvement in the field or analytical laboratory work done for the 16 Cities Study and/or tobacco industry sponsorship of the study, and that although we ultimately did publish papers regarding the impact of smoking restrictions on workplace exposures, those papers were “published long after the close of the OSHA proceedings, ….” (Barnes et al. 2006, p. 1896). Although the authors cited my 5 January 1995 OSHA testimony, they did not mention the ≥1,000-word explanation of the role of RJ Reynolds and Bellomy Research in the 16 Cities Study in my formal presentation to OSHA (OSHA 1995). Neither did they mention the discussions during my questioning regarding the work flow in the study or the pages of discussions regarding the contractual arrangements between Martin Marietta Energy Systems [Oak Ridge National Laboratory’s (ORNL) prime contractor at the time] and the Center for Indoor Air Research, the study’s sponsor. More importantly, in the first article on the 16 Cities Study (Jenkins et al. 1996; p. 475), a paragraph is devoted to the tasks and responsibilities of the three institutions (ORNL, RJ Reynolds, and Bellomy Research). On pages 480, 481, and 483, there is a detailed discussion of the work flow, quality control, and data inspection among the three institutions. Finally, on page 500, we acknowledged the funding source for the study and the contributions of key individuals from the other two institutions. Did Barnes et al. (2006) just miss the aforementioned discussions, or did they ignore them?

As to the issue of whether OSHA was made aware of the impact of workplace smoking in a timely fashion, the contention of Barnes et al. (2006) is false. In fact, as part of its deliberations, OSHA conducted expert workshops on exposure assessment, health effects, and ventilation beginning in September 1997. Both S.K. Hammond and I, along with others, were invited participants of the workshop on exposure assessment held at Johns Hopkins University on 12–13 September 1997. At that workshop, I provided a variety of analyses to the panel, including one concerning the impact of smoking restrictions on personal exposures to ETS in the workplace. That analysis was eventually published (Jenkins and Counts 1999). For Barnes et al. to claim that we did not make OSHA aware of the impact of workplace smoking restrictions on exposure before the close of the proceedings, when one of the authors was present at an OSHA expert workshop that was part of OSHA’s “proceedings” where I presented the data analyses in question, is both astounding and false.

The 16 Cities Study stands as the largest and most representative study of personal exposure to ETS ever conducted in the United States. The methodology used was sound and the findings scientifically valid. Seven peer-reviewed papers have been published from its work, including one in *Environmental Health Perspectives*. If Barnes et al. (2006) disagree with the manner in which we organized or interpreted the data in those seven articles, I would point out that a flat-field version of our 16 Cities Study results database has been available to the public since at least 2000 through either the Sapphire Group (Gevecker Graves et al. 2000), or from the Oak Ridge National Laboratory’s web site (Oak Ridge National Laboratory 2005). We have always welcomed fresh eyes on our data, and we are disappointed that Barnes et al. appear not to have taken advantage of its availability.

Science is about a dispassionate analysis of the facts—all the facts. All of the facts must be analyzed, even if they do not support a hypothesis or preconceived outcome of the analysis. Anything less can be considered poor science or, at worst, politics.
